# External validation of the NeuroImaging Radiological Interpretation System and Helsinki computed tomography score for mortality prediction in patients with traumatic brain injury treated in the intensive care unit: a Finnish intensive care consortium study

**DOI:** 10.1007/s00701-022-05353-0

**Published:** 2022-09-01

**Authors:** Juho Vehviläinen, Markus Skrifvars, Matti Reinikainen, Stepani Bendel, Ruut Laitio, Sanna Hoppu, Tero Ala-Kokko, Jari Siironen, Rahul Raj

**Affiliations:** 1 Department of Neurosurgery, Helsinki University Hospital and University of Helsinki, Topeliuksenkatu 5, P.B. 266, 00029 HUS Helsinki, Finland; 2 Department of Emergency Care and Services, University of Helsinki and Helsinki University Hospital, Helsinki, Finland; 3 Department of Anesthesiology and Intensive Care, Kuopio University Hospital & University of Eastern Finland, Kuopio, Finland; 4 Department of Perioperative Services, Intensive Care and Pain Management, Turku University Hospital & University of Turku, Turku, Finland; 5 Department of Intensive Care and Emergency Medicine Services, Department of Emergency, Anesthesia and Pain Medicine, Tampere University Hospital & University of Tampere, Tampere, Finland; 6 Research Group of Surgery, Anesthesiology and Intensive Care, Division of Intensive Care, Medical Research Center, Oulu University Hospital & University of Oulu, Oulu, Finland

**Keywords:** Traumatic brain injury, Intensive care, Computed tomography, Trauma surgery

## Abstract

**Background:**

Admission computed tomography (CT) scoring systems can be used to objectively quantify the severity of traumatic brain injury (TBI) and aid in outcome prediction. We aimed to externally validate the NeuroImaging Radiological Interpretation System (NIRIS) and the Helsinki CT score. In addition, we compared the prognostic performance of the NIRIS and the Helsinki CT score to the Marshall CT classification and to a clinical model.

**Methods:**

We conducted a retrospective multicenter observational study using the Finnish Intensive Care Consortium database. We included adult TBI patients admitted in four university hospital ICUs during 2003–2013. We analyzed the CT scans using the NIRIS and the Helsinki CT score and compared the results to 6-month mortality as the primary outcome. In addition, we created a clinical model (age, Glasgow Coma Scale score, Simplified Acute Physiology Score II, presence of severe comorbidity) and combined clinical and CT models to see the added predictive impact of radiological data to conventional clinical information. We measured model performance using area under curve (AUC), Nagelkerke’s R^2^ statistics, and the integrated discrimination improvement (IDI).

**Results:**

A total of 3031 patients were included in the analysis. The 6-month mortality was 710 patients (23.4%). Of the CT models, the Helsinki CT displayed best discrimination (AUC 0.73 vs. 0.70 for NIRIS) and explanatory variation (Nagelkerke’s R^2^ 0.20 vs. 0.15). The clinical model displayed an AUC of 0.86 (95% CI 0.84–0.87). All CT models increased the AUC of the clinical model by + 0.01 to 0.87 (95% CI 0.85–0.88) and the IDI by 0.01–0.03.

**Conclusion:**

In patients with TBI treated in the ICU, the Helsinki CT score outperformed the NIRIS for 6-month mortality prediction. In isolation, CT models offered only moderate accuracy for outcome prediction and clinical variables outweighing the CT-based predictors in terms of predictive performance.

**Supplementary Information:**

The online version contains supplementary material available at 10.1007/s00701-022-05353-0.

## Introduction

Traumatic brain injury (TBI) is one of the most common causes of mortality among young persons [3, 4]. In recent years, it has been identified as an increasing risk of mortality and morbidity among elderly as well [22]. Glasgow Coma Scale (GCS) has been traditionally used as a measure of TBI severity upon admission to hospital. GCS is easy and fast to assess; however, it does not give information on structural information on potential intracranial lesions.

As computed tomography (CT) has become widely available, several classifications and scoring systems have been developed for additional information on TBI prognosis. These include, e.g., Marshall CT classification [8], Rotterdam CT-based score [7], Helsinki CT score [17], and Stockholm CT score [12].

The practical use of CT scores is to give clinicians more quantitative and comparable tools to assess the severity of TBI and estimate need for operative treatment and prognosis. For research purposes, CT scores are used for injury severity standardization and comparison. Recently, a new CT score, NeuroImaging Radiological Interpretation System (NIRIS), was introduced [25]. The NIRIS consists of five categories ranging from 0 to 5, with an increasing intracranial injury load with an increasing number. NIRIS was developed to consolidate imaging findings into different categories of ordinal severity to inform specific patient management actions [25]. The NIRIS has been earlier validated against Marshall CT classification and Rotterdam CT score [2, 25, 27]. To our knowledge, NIRIS has not been validated nor compared to more granular Helsinki CT score.

In this study, we aimed to perform an external validation study of the NIRIS and to compare it with the Helsinki CT score and a clinical model for predicting 6-month mortality. We hypothesized that both CT scores would add predictive performance when compared just with clinical data and that the Helsinki CT score would outperform the NIRIS, as it is more granular. We also report performance statistics of the widely used Marshall CT classification system.

## Methods and materials

The ethics committee of Helsinki University Hospital (194/13/03/14 §97), the Finnish National Institute for Health and Welfare (THL/713/5.05.01/2014 and THL/1298/5.05.00/2019), Statistics Finland (TK-53–1047-14), the Office of the Data Protection Ombudsman (Dnro 2713/402/2016 28.10.16), and all the participating university hospitals’ research committees approved this study. The study adhered to the Strengthening the Reporting of Observational Studies in Epidemiology (STROBE) guidelines.

### Study design and population

We performed a multicenter retrospective observational study using data that were prospectively collected from the Finnish Intensive Care Consortium (FICC) database. The FICC database is a nationwide prospectively data-collecting database including all ICU-treated patients from the majority of all ICUs in Finland [19]. In Finland, all specialized tertiary intensive care of TBI patients is centralized to five tertiary ICUs. Four of these ICUs participate in the FICC covering approximately two-thirds of the population in Finland. From these four tertiary ICUs, we included all adult TBI patients (age ≥ 18 years) admitted from January 1, 2003, to December 31, 2013 (readmissions excluded). Patients were excluded if no primary CT scan was available, and if Glasgow Coma Scale (GCS) or pre-admission functional status was missing.

Six-month case fatality was used as endpoint for analysis (available for all Finnish citizens through the Finnish population registry).

### CT assessment

All patients in the study had non-contrast CT scan taken at the admission to hospital. Patients with only post-operative CT scans, CT angiography, or MRI scans were excluded. All available CT images were classified according to the Marshall CT classification system, the Helsinki CT score, and the updated version of NIRIS [27] by two authors (JV, RR). The CT classification systems are described in Table 1.

**Table 1:** Description of Marshall CT classification, Helsinki CT score, and NIRIS methods

CT classification/scoring system	Classification or component	Description
Marshall CT classification	Diffuse injury I	No visible intracranial pathology
	Diffuse injury II	Basal cisterns are present with midline shift 0–5 mm and no high- or mixed-density lesions > 25 cm^3^
	Diffuse injury III	Basal cisterns compressed or absent, midline shift 0–5 mm, no high- or mixed-density lesion > 25 cm^3^
	Diffuse injury IV	Midline shift > 5 mm, no high- or mixed-density lesion > 25 cm^3^
	EML V/NEMLVI	High- or mixed-density lesions > 25 cm^3^
Helsinki CT score	Mass lesion type	SDH: 2 ICH or contusion: 2 EDH: -3
	Mass lesion size	Haematoma volume > 25 cm^3^: 2
	IVH	Absent: 0, present: 3
	Suprasellar cisterns	Normal: 0, compressed: 1, obliterated: 5
	Sum score	Range: − 3 to 14
NIRIS	Category 0	No abnormal finding
	Category 1	Fracture Pneumocephalus* EDH, SDH, ICH, or parenchymal contusion < 0.5 cm^3^ Subarachnoid hemorrhage
	Category 2	EDH, SDH, ICH, or parenchymal contusion > 0.5 cm^3^ Diffuse axonal injury IVH Mild or moderate hydrocephalus* Midline shift 0–5 mm
	Category 3	EDH, ICH, or parenchymal contusion > 15 cm^3^ SDH > 50 cm3 Midline shift > 5 mm Focal herniation
	Category 4	EDH, ICH, or parenchymal contusion > 20 cm^3^ SDH > 200 cm^3^* Severe hydrocephalus Midline shift > 10 mm Diffuse herniation Duret hemorrhage

*Abbreviations: EML,* evacuated mass lesion; *NEML,* non-evacuated mass lesion; *EDH,* epidural haematoma; *NIRIS,* NeuroImaging Radiological Interpretation System; *SDH,* subdural haematoma; *ICH,* intracerebral haematoma (parenchymal); *IVH,* intraventricular hemorrhage; *The revised NIRIS definitions^9^

### Statistical analysis

The Marshall CT classification [8] and the NIRIS [25, 27] were treated as categorical variables, NIRIS being ordinal. The Helsinki CT score was originally constructed as an ordinal scale, but due to its many levels and numeric distribution, it can be treated as a numeric variable [17].

We performed first-level customization of the CT scores by fitting a new logit function to the respective CT score [10]. The Helsinki CT-based score (hereafter referred to as Helsinki CT score), Marshall CT-based classification (hereafter referred to as Marshall CT class), and NIRIS-based (hereafter referred to as NIRIS).

We created a clinical “base model” that included age, GCS score (worst measured GCS score during the first ICU-day or as the last reliable GCS for intubated and/or sedated patients), a modified Simplified Acute Physiology Score II (SAPS II, without the age, GCS score, and chronic comorbidity component), and the presence of a chronic comorbidity (according to the SAPS II and Acute Physiology and Chronic Evaluation [APACHE] II definitions). The SAPS II and APACHE scores were assessed during the first 24 h of ICU treatment. We separately added age, GCS score, and chronic comorbidities to give them more weight in our base model [18]. To the base model, the three different CT scores (NIRIS, Marshall CT classification, and Helsinki CT score) were separately added.

We assessed the individual CT scores and the combined base + CT scores by calculating the Nagelkerke’s R^2^ and the area under the receiver operating characteristics curve [11]. Nagelkerke’s R^2^ gives a value between 0 and 1 resembling explained variance, where the value 1 indicates a model that fully explains the outcome. The AUC values range from 0.5 to 1 with 0.5 indicating at the level of chance and 1 indicating a perfect model. We assessed the calibration by using the Hosmer–Lemeshow test for all models apart from NIRIS and Marshall, as these consist of less than 10 groups.

We compared AUCs between models using a DeLong test. We considered p-values under 0.05 statistically significant.

In addition to the AUC analysis, we calculated the integrated discrimination improvement (IDI) as the AUC might be more insensitive in model comparisons in which the baseline model has performed well [14]. The IDI is a category free measure of the discrimination ability between two logistic regression prediction models. The IDI can be defined as the difference in discrimination slopes between two models one with, and the other without, the added variable. It can be estimated with the following equation: $\widehat{IDI}=\left({\overline{\widehat{p}}}_{new,events}-{\overline{\widehat{p}}}_{old,events}\right)-\left({\overline{\widehat{p}}}_{new,nonevents}-{\overline{\widehat{p}}}_{old,nonevents}\right)$, where ${\overline{\widehat{p}}}_{new,events}$ is the mean of the new model-based predicted probabilities of an event for those who develop events, ${\overline{\widehat{p}}}_{old,events}$ is the corresponding quantity based on the old model, ${\overline{\widehat{p}}}_{new,nonevents}$ is the mean of the new model-based predicted probabilities of an event for those who do not develop events, and ${\overline{\widehat{p}}}_{old,nonevents}$ is the corresponding quantity based on the old model [13]. Another representation of the IDI can be formulated with the following equation: $IDI=\left({IS}_{new}-{IS}_{old}\right)-({IP}_{new}-{IP}_{old})$, where $IS= \int sensitivity$ and $IP= \int 1-specificity$ and the subscripts “new” and “old” correspond to the new model and the old model, respectively [13]. The IDI reflects the mean magnitude of the change in outcome probability with the addition of the new variable in the prediction model. It is the area between the curves (the old model and the new model) in a plot where on the y-axis are both sensitivity and 1-specificity, and on the x-axis is the calculated risk. The model performance is improved when the new model moves the reference curve of sensitivity toward the top-right corner and the reference curve of 1-specificity toward lower-left corner. Conversely, model performance is reduced when the new model moves the reference curve of sensitivity toward lower-left corner and the reference curve of 1-specificity toward top-right corner [16, 24]. The IDI ranges theoretically from − 2 to 2 representing the overall risk discrimination improvement [13, 15, 16, 21].

We used SPSS IBM Corp. Released 2020. IBM SPSS Statistics for Windows, Version 27.0. Armonk, NY: IBM Corp and STATA StataCorp. 2019. Stata Statistical Software: Release 16. College Station, TX: StataCorp LLC for the statistical analysis.

## Results

**Fig. 1:**
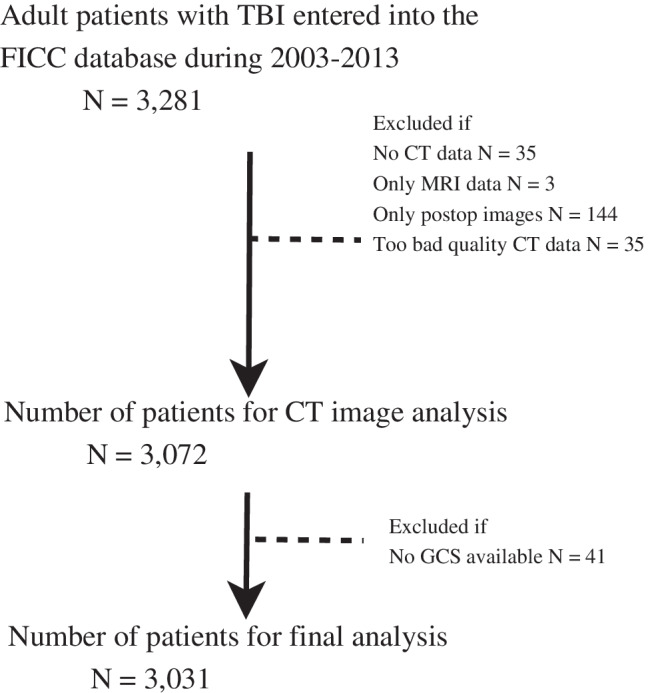
Study’s patient flow chart. Abbreviations: CT = computed tomography; MRI = magnetic resonance imaging; GCS = Glasgow Coma Scale; FICC = Finnish Intensive Care Consortium; TBI = traumatic brain injury. Note that some CT images had more than one exclusion criteria

Altogether, 3031 patients met the inclusion criteria (Fig. 1). Patient characteristics are listed in Table 2. The median patient age was 55 years, 78% were male, 90% were functionally independent prior to admission, and 8.3% suffered from significant comorbidity. Almost half (47%) had a GCS between 3 and 8 in the first 24 h or before intubation. Approximately one-third (33%) of the patients required operative treatment, 24% were ICP monitored, and majority of the patients (66%) were intubated and mechanically ventilated. Seven percent of patients died during ICU treatment, 13% died in hospital, and 23% in 6 months after the TBI.

**Table 2:** Patient characteristics

Variables	All patients N = 3031
Age (yr.), median (IQR)	55 (41, 67)
18–40, N (%)	753 (24.8%)
1–64, N (%)	1419 (46.8%)
≥ 65, N (%)	859 (28.3%)
Glasgow Coma Scale score, median (IQR)	9.0 (5.0, 14.0)
3–8, N (%)	1410 (46.5%)
9–12, N (%)	585 (19.3%)
13–15, N (%)	1036 (34.2%)
Females, N (%)	676 (22.3%)
Preadmission performance status^1^, N (%)*	
Fit for work or equal	1827 (60.3%)
Unfit for work, but independent in self-care	908 (30.0%)
Partially dependent in self-care	174 (5.7%)
Totally dependent in self-care	48 (1.6%)
Significant chronic comorbidity^2^, N (%)	252 (8.3%)
Operative admission, N (%)†	994 (32.8%)
Mechanical ventilation, N (%)	1990 (65.7%)
Intracranial pressure monitoring, N (%)	711 (23.5%)
LOS university neurosurgical ICU (d), median (IQR)	1.63 (0.79, 3.84)
LOS university hospital (d), median (IOR)	6.00 (3.00, 11.0)
Acute Physiological and Chronic Health Evaluation II score (APACHE), median (IQR)	18.00 (12.00, 24.00)
Simplified Acute Physiology Score II, median (SAPS II) (IQR)	34.00 (23.00, 50.00)
Sequential Organ Failure Assessment score (SOFA), median (IQR) ‡	6.00 (3.00, 8.00)
Outcome	
Death at ICU, N (%)	220 (7.3%)
Death during hospitalization, N (%) §	389 (12.8%)
Dead at 6 months from TBI, N (%)	710 (23.4%)

*Abbreviations: IQR*, interquartile range; *LOS*, length of stay; *NIRIS*, NeuroImaging Radiological Interpretation System. ^1^A modified World Health Organization/Eastern Cooperative Oncology Group classification system implemented by the Finnish Intensive Care Consortium. ^2^Any chronic comorbidity according to Acute Physiology and Chronic Health Evaluation II or Simplified Acute Physiology Score II. Missing values (N (%)): *74 (2.4%); † 2 (0.1%); ‡ 6 (0.2%); § 2 (0.1%)

 The distribution of NIRIS categories, Marshall CT classes, and median Helsinki CT scores is shown in Table 3. NIRIS category 2 (43%) was the most frequent, followed by NIRIS category 4 (22%) and 3 (17%). The most frequent Marshall classes were EML/NEML (44%) and II (43%). The median Helsinki CT score was 2.0 (IQR 2.0–4.0). NIRIS categories 0–1 correlated well with Marshall classes I and II. Most patients with a NIRIS category of II had a Marshall class of II. The majority of patients with a NIRIS category of 3 and 4 had a Marshall class indicating mass lesion (EML/NEML).

**Table 3:** Number of patients in NIRIS categories and corresponding Marshall CT classifications and medians of Helsinki CT score

	NIRIS category				
	0	1	2	3	4
N (%)	320 (10.6%	225 (7.4%)	1297 (42.8%)	526 (17.4%)	663 (21.9%)
Corresponding values for Marshall CT classification and Helsinki CT score					
Marshall CT classification N (%)*					
I	319 (99.7%)	14 (6.2%)	6 (0.4%)	0	0
II	1 (0.3%)	208 (92.5%)	739 (57.0%)	72 (13.7%)	7 (1.1%)
III	0	3 (1.3%)	232 (17.9%)	54 (10.3%)	4 (0.6%)
IV	0	0	2 (0.2%)	28 (5.3%)	18 (2.7%)
EML V/NEML VI	0	0	318 (24.5%)	372 (70.7%)	634 (95.6%)
Helsinki CT score, median (IQR)	0.0 (0.0, 0.0)	2.0 (0.0, 2.0)	2.0 (2.0, 4.0)	4.0 (2.0, 4.0)	5.0 (4.0, 9.0)

^*^% within NIRIS category

Non-survivors and patients with a low GCS score 3–8 in the first 24 h, or before intubation, more often belonged to a higher NIRIS category than survivors (Table 4). The median age, frequency of mechanical ventilation, operative treatment, and ICP monitoring increased with a rising NIRIS category (Table 4). When divided further into groups of NIRIS categories and respective GCS groups (3–8; 9–12; 13–15), the correlation between ICP monitoring and increasing NIRIS category was not as clear (Supplemental Table 1). The 6-month mortality in patients with NIRIS categories 0, 1, 2, 3, and 4 was 8.4%, 5.3%, 17.0%, 27.0%, and 46.6%, respectively.

**Table 4:** Association between NIRIS category, age, GCS, treatment-related factors, and mortality

	NIRIS category					
	0	1	2	3	4	Total
N (%)	320 (10.6%)	225 (7.4%)	1297 (42.8%)	526 (17.4%)	663 (21.9%)	3031 (100%)
Age, median (IQR)	38 (26, 54)	43 (25, 57)	54 (41, 67)	59 (47, 69)	60 (51, 70)	55 (41, 67)
Glasgow Coma Scale score, median (IQR)						
3–8, N (%*)	104 (32.5%)	77 (34.2%)	512 (39.5%)	240 (45.6%)	477 (71.9%)	1410 (46.5%)
9–12, N (%*)	52 (16.3%)	37 (16.4%)	268 (20.7%)	138 (26.2%)	90 (13.6%)	585 (19.3%)
13–15, N (%*)	164 (51.2%)	111 (49.3%)	517 (39.9%)	148 (28.1%)	96 (14.5%)	10,136 (34.2%)
Mechanical ventilation, N (%*)	177 (55.3%)	119 (52.9%)	747 (57.6%)	370 (70.3%)	577 (87.0%)	1990 (65.7%)
Operative treatment, N (%*)	8 (2.5%)	6 (2.7%)	263 (20.3%)	273 (52.0%)	444 (67.1%)	994 (32.8%)
Intracranial pressure monitoring, N (%*)	36 (11.3%)	45 (20.0%)	317 (24.4%)	143 (27.2%)	170 (25.6%)	711 (23.5%)
Dead ICU, N (%*)	9 (2.8%)	2 (0.9%)	63 (4.9%)	38 (7.2%)	108 (16.3%)	220 (7.3%)
Dead hospital, N (%*)	19 (5.9%)	6 (2.7%)	105 (8.1%)	72 (13.7%)	187 (28.2%)	389 (12.8%)
Dead 6-months, N (%*)	27 (8.4%)	12 (5.3%)	220 (17.0%)	142 (27.0%)	309 (46.6%)	710 (23.4%)

^*^% within NIRIS category. According Chi-Square test, all variables were statistically significantly (p < 0.01) dependent. Abbreviations: *IQR*, interquartile range; *NIRIS*, NeuroImaging Radiological Interpretation System

### Performance of the models

**Fig. 2:**
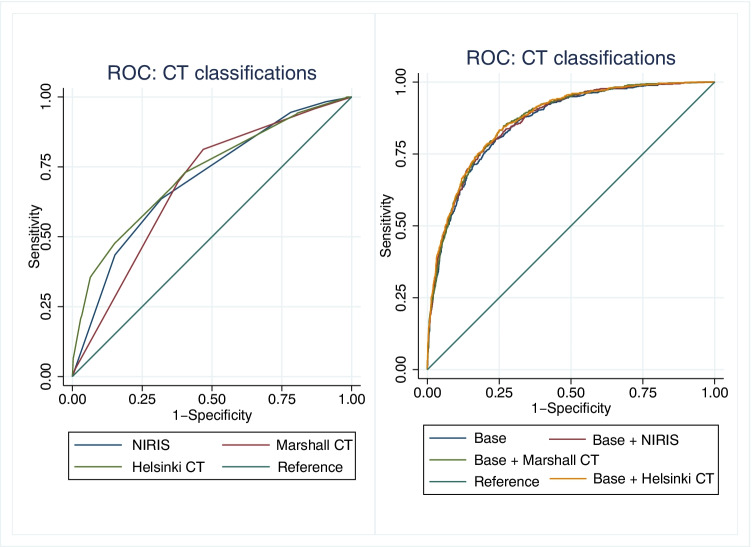
On the left: ROC curves of NIRIS, Marshall CT classification, and Helsinki CT score vs. 6-month mortality. On the right: ROC curves of NIRIS, Marshall CT classification, and Helsinki CT score combined to a base model vs. 6-month mortality

Of the CT models, Helsinki CT displayed best discrimination (AUC 0.73 vs. 0.70 for NIRIS vs. 0.68 for Marshall, Fig. 2) and explained variance (Nagelkerke’s R^2^ 0.20 vs. 0.15 for NIRIS vs. 0.14 for Marshall) (Table 5).

**Table 5:** Performance measures of the CT scores, base model, and combined models for predicting 6-month mortality

CT models	AUC (95% CI)	p value^ǂ^	Hosmer–Lemeshow p value	Nagelkerke’s R^2^	IDI (95% CI)
NIRIS	0.70 (0.68–0.72)	Ref	NA	0.147	NA
Marshall CT	0.68 (0.66–0.70)	0.021	NA	0.136	NA
Helsinki CT	0.73 (0.70–0.75)	0.007	0.20	0.200	NA
Clinical + CT models					
Base^*^	0.86 (0.84–0.87)	Ref	0.96	0.427	Ref
Base + NIRIS	0.87 (0.85–0.88)	< 0.001	0.51	0.442	0.012 (0.007–0.017)
Base + Marshall CT	0.87 (0.85–0.88)	< 0.001	0.95	0.448	0.011 (0.006–0.016)
Base + Helsinki CT	0.87 (0.86–0.88)	< 0.001	0.71	0.456	0.028 (0.019–0.036)

^*^Based on age, GCS, chronic comorbidities, and modified SAPS II. ǂ AUC comparison using DeLong test to NIRIS for CT models and to Base model for Clinical + CT models. Abbreviations: *AUC*, area under curve; *CT*, computed tomography; *GCS*, Glasgow Coma Scale; *IDI*, integrated discrimination improvement; *NIRIS*, NeuroImaging Radiological Interpretation; *SAPS*, Simplified Acute Physiology Score. The Hosmer–Lemeshow test was not applicable for NIRIS and Marshall CT as these consist of less than 10 categories

The base model displayed an AUC of 0.86 (95% CI 0.84–0.87) with an explained variance of 0.43. The addition of all CT models increased the AUC by + 0.01 to 0.87 (p < 0.001, Table 5, Fig. 2). The explained variance increased to 0.44 when adding the NIRIS to the base model and to 0.46 when adding the Helsinki CT score to the base model. The IDI values were positive (ranging from 0.011 to 0.028) for all CT models when they were added to the base model.

## Discussion

### Key findings

In this large multicenter observational study, including 3031 patients from four academic centers in Finland, we compared three different CT scores to their ability to predict 6-month mortality in patients with TBI treated in the ICU. Of the three CT models in isolation, the Helsinki CT score displayed the best performance for 6-month mortality prediction. However, after adding the individual CT models to a clinical base model, only a moderate improvement in predictive performance predicting the 6-month mortality could be seen: AUC by + 0.01 to 0.87 (95% CI 0.85–0.88) and the IDI by 0.01–0.03, which is small, but statistically significant, and makes the AUC analysis more robust. Our results suggest that the choice of CT model when adjusting for case-mix in patients with TBI is of less importance than the adjustment of clinical variables such as age, GCS score, comorbidities, and acute physiological derangements (e.g., SAPS II).

### Comparison to previous studies

The NIRIS has been validated in a more general TBI population and compared to the Marshall CT classification and the Rotterdam CT score [2, 27]. Their results showed that the NIRIS performed similarly to the Marshall CT classification and the Rotterdam CT score in terms of predicting mortality, but markedly better in terms of discriminating the needed interventions and intensity of patient care. This is in line with the original aim of the NIRIS to predict TBI patient care based on initial imaging.

The study populations in both original article introducing the NIRIS [25] and in the validation article by Zhou et al., [27] done in Stanford Hospital, USA, had a more general TBI population compared to our already ICU-admitted study population. Hence, our study population had more severe injuries and higher mortality: approx. 2% in earlier studies with the NIRIS compared to almost 13% during hospitalization in our study. This is also seen in the NIRIS categories as category 2 is the most common (43%) in our study population compared to category 0 in Zhou et al. [27] (77%). Furthermore, categories 3 and 4 include 39% of our study population that leaves less than 20% for categories 0 and 1. A validation study [2] of NIRIS conducted in India had a similar mortality rate in hospital (14%) than in our study; however, the patients were general TBI patients, not only ICU-admitted TBI patients.

In contrast to the NIRIS, the Helsinki CT score was developed for outcome prediction in patients with TBI treated in the ICU [17]. The Helsinki CT score has been validated in pediatric TBI patients (AUC 0.84) [9], penetrating TBI patients (AUC 0.90) [6], and adult TBI patients (AUC 0.70–0.81) [1, 5, 20, 23, 26] with good performance. We found similar performance measures in the present cohort (AUC 0.73, Nagelkerke’s R^2^ 0.20). Thus, due to differences in design and granularity, it is not surprising that the Helsinki CT score outperformed the NIRIS in terms of outcome prediction. Furthermore, in line with previous results, clinical variables seem to be more important predictors that CT predictors [18].

The clinical importance of early CT imaging in patients with significant TBI is undisputed. However, current CT models seem to be of limited additional prognostic value compared to clinical variables in terms of mortality prediction. There is some obvious selection bias to this, as all patients were admitted to the ICU. Thus, the association between variables such as midline shift and mass lesions is diluted since these may be, at least partly, reversible due to surgical treatment [23]. It is possible that the predictive performance of the current CT models could be improved by including spatial and volumetric parameters.

The SAPS II and APACHE scores are measured during the first 24 h of ICU treatment and thus contain more information than the CT models based upon admission characteristics. This will be in favor of the clinical model in terms of prognostic accuracy.

### Strengths and limitations

Some strengths should be highlighted. We used a large multicenter high-quality database collecting data prospectively. Thus, we were able to include more than three thousand patients in our study. In addition, there was a small number of missing data, and we had a complete 6-month follow-up. Our patient cohort also represents well the general ICU-treated TBI population in Finland as the referral population of the four neurointensive ICUs is approximately 3.5 million people, encompassing two-thirds of the Finnish population.

Some limitations should be acknowledged. The FICC is a general ICU database and lacks some TBI-specific parameters, like specific neurosurgical procedures, admission GCS score, pupillary light reactivity, and thus information on IMPACT or CRASH models. The FICC database used in this study did not include data on such devastating injuries that the more aggressive treatment was withheld. Information regarding admission due to organ donation has been added later. Still, for case-mix adjustment, our base model displayed good statistical performance. Second, we only had all-cause mortality as an outcome measure. Predicting functional outcome would be desirable as well. Noteworthy, the Helsinki CT score was designed to predict outcome while Marshall CT and NIRIS were not. Thus, this might skew the results in favor of the Helsinki CT score when predicting outcome. Third, we highlight that we only included patients treated in a university hospital ICU and did not include milder TBIs. In this study, we did not include in comparisons the two other significant CT scores, namely, the Stockholm CT score and the Rotterdam CT score. Earlier, both of these scores have been validated against the Helsinki CT score [23] and the Rotterdam CT score against the NIRIS [2, 27]. The Stockholm CT score has shown to have superior predictive power, to some extent, over the Helsinki CT score and the Rotterdam CT score [23]. Further work should be done to compare the Stockholm CT score to the NIRIS.

## Conclusion

In patients with TBI treated in the ICU, the Helsinki CT score outperformed the NIRIS for 6-month mortality. However, clinical variables outweighed the current CT-based models in terms of predictive performance. Thus, accounting for clinical variables when adjusting for TBI injury severity is imperative.

## Supplementary Information

Below is the link to the electronic supplementary material.

Supplementary file1 (DOCX 15 KB)
