# Supplementary material for: External validation of the NeuroImaging Radiological Interpretation System and Helsinki computed tomography score for mortality prediction in patients with traumatic brain injury treated in the intensive care unit: a Finnish intensive care consortium study
Source: Acta Neurochir (Wien). 2022 Sep 1;164(10):2709–17. doi: 10.1007/s00701-022-05353-0 (PMC9519640; doi:10.1007/s00701-022-05353-0)
Supplement: Supplementary file 1 — Supplementary file1 (DOCX 15 KB) [file 701_2022_5353_MOESM1_ESM.docx]

**External validation of the NeuroImaging Radiological Interpretation System and Helsinki Computed Tomography score for Mortality Prediction in Patients with Traumatic Brain Injury Treated in the Intensive Care Unit: A Finnish Intensive Care Consortium Study**

Acta Neurochirurgica

Juho Vehviläinen*^1^, MD, M.Sc.; Markus Skrifvars^2^, MD, PhD; Matti Reinikainen^3^, MD, PhD; Stepani Bendel^3^, MD, PhD; Ruut Laitio^4^, MD, PhD; Sanna Hoppu^5^, MD, PhD; Tero Ala-Kokko^6^, MD, PhD; Jari Siironen^1^, MD, PhD; Rahul Raj^1^, MD, PhD,

^1^Department of Neurosurgery, Helsinki University Hospital and University of Helsinki, Helsinki, Finland

^2^Department of Emergency Care and Services, University of Helsinki and Helsinki University Hospital, Helsinki, Finland

^3^Department of Anesthesiology and Intensive Care, Kuopio University Hospital & University of Eastern Finland, Kuopio, Finland

^4^Department of Perioperative Services, Intensive Care and Pain Management, Turku University Hospital & University of Turku, Turku, Finland

^5^Department of Intensive Care and Emergency Medicine Services, Department of Emergency, Anesthesia and Pain Medicine, Tampere University Hospital & University of Tampere, Tampere, Finland

^6^Research Group of Surgery, Anesthesiology and Intensive Care, Division of Intensive Care, Oulu University Hospital & University of Oulu, Medical Research Center, Oulu, Finland

Corresponding author*: **Juho Vehviläinen**

- E-mail: juho.vehvilainen@helsinki.fi

Supplemental Table 1: Number of patients with intracranial pressure monitoring and operative treatment divided by different GCS groups and NIRIS categories.

|  | NIRIS Category | | | | |
| --- | --- | --- | --- | --- | --- |
|  | 0 | 1 | 2 | 3 | 4 |
| N (%) | 320 (10.6%) | 225 (7.4%) | 1297 (42.8%) | 526 (17.4%) | 663 (21.9%) |
| **GCS 3-8, N (%*)** | 104 (32.5%) | 77 (34.2%) | 512 (39.4%) | 240 (45.6%) | 477 (71.9%) |
| Intracranial pressure monitoring, N (%**) | 26 (25.0%) | 36 (46.8%) | 240 (46.9%) | 108 (45.0%) | 139 (29.1%) |
| Operative treatment, N (%**) | 3 (2.9%) | 4 (5.2%) | 117 (22.9%) | 118 (49.4%) | 318 (66.8%) |
| **GCS 9-12, N (%*)** | 52 (16.3%) | 37 (16.4%) | 268 (20.7%) | 138 (26.2%) | 90 (13.6%) |
| Intracranial pressure monitoring, N (%**) | 5 (9.6%) | 7 (18.9%) | 49 (18.3%) | 24 (17.4%) | 20 (22.2%) |
| Operative treatment, N (%**) | 3 (5.8%) | 1 (2.7%) | 58 (21.6%) | 80 (58.0%) | 64 (71.1%) |
| **GCS 13-15, N (%*)** | 164 (51.3%) | 111 (49.3%) | 517 (39.9%) | 148 (28.1%) | 96 (14.5%) |
| Intracranial pressure monitoring, N (%**) | 5 (3.0%) | 2 (1.8%) | 28 (5.4%) | 11 (7.4%) | 11 (11.5%) |
| Operative treatment, N (%**) | 2 (1.2%) | 1 (0.9%) | 88 (17.0%) | 75 (50.7%) | 62 (64.6%) |
| *% within NIRIS category  **% within NIRIS and GCS category  Abbreviations: GCS = Glasgow Coma Score; NIRIS = NeuroImaging Radiological Interpretation System | | | | | |
